# Versatile Chemical Derivatizations to Design Glycol Chitosan-Based Drug Carriers

**DOI:** 10.3390/molecules22101662

**Published:** 2017-10-05

**Authors:** Sung Eun Kim, Hak-Jun Kim, Jin-Kyu Rhee, Kyeongsoon Park

**Affiliations:** 1 Department of Orthopedic Surgery and Rare Diseases Institute, Korea University Medical College, Guro Hospital, Seoul 08308, Korea; sekim10@korea.ac.kr (S.E.K.); dakjul@korea.ac.kr (H.-J.K.); 2 Department of Food Science and Engineering, Ewha Womans University, Seoul 03760, Korea; 3 Department of Systems Biotechnology, College of Biotechnology and Natural Resources, Chung-Ang University, Gyeonggi-do 17546, Korea

**Keywords:** glycol chitosan, chemical derivatizations, nanoparticles, drug carriers, disease therapy

## Abstract

Glycol chitosan (GC) and its derivatives have been extensively investigated as safe and effective drug delivery carriers because of their unique physiochemical and biological properties. The reactive functional groups such as the amine and hydroxyl groups on the GC backbone allow for easy chemical modification with various chemical compounds (e.g., hydrophobic molecules, crosslinkers, and acid-sensitive and labile molecules), and the versatility in chemical modifications enables production of a wide range of GC-based drug carriers. This review summarizes the versatile chemical modification methods that can be used to design GC-based drug carriers and describes their recent applications in disease therapy.

## 1. Introduction

Advanced nanotechnologies have greatly contributed to the development of various types of nanodrug carriers, including liposomes, polymeric nanoparticles, and inorganic nanovehicles [1]. These drug carriers sufficiently accumulate in diseased tissues where there is leaky vasculature and poor lymphatic drainage (termed enhanced permeability and retention (EPR) effects) [2]. Through passive tumor-homing based on EPR effects, systemically-delivered drug carriers can ferry various types of drugs including small drugs, peptides/proteins, and nucleic acids and are highly effective in enhancing the therapeutic efficacies of drugs while reducing their side effects [3,4]. Additionally, nano-sized drug carriers with signal emitters (e.g., fluorophores, isotopes, and magnetic nanoparticles) can be used to sense pathophysiological defects and to monitor or predict therapeutic responses [5]. 

A wide range of synthetic or natural polymers have been investigated for drug delivery systems [6]. Among them, polysaccharides have received increasing attention in biomedical research because of their biocompatibility, biodegradability, low toxicity, and low cost [7]. Chitosan is a nontoxic, low-immunogenic, biodegradable, and biocompatible linear polysaccharide comprising copolymers of glucosamine and *N*-acetylglucosamine [8]. Typically, chitosan can be derived by deacetylation of the *N*-acetyl glucosamine units of chitin from crustacean shells, generally by hydrolysis under alkali conditions at high temperature [9]. The term chitosan is used to describe a series of chitosan polymers (e.g., different molecular weights ranged from 5.0 × 10^4^ to 2.0 × 10^6^, viscosity (1% chitosan in 1% acetic acid, <2000 mPa·S), and degree of deacetylation (40–98%)) [10]. Due to its remarkable biological properties, chitosan has been widely applied to the pharmaceutical and biomedical fields for drug delivery [11], tissue engineering [12], and inhibition of bacterial infection [13]. However, chitosan is normally insoluble in water above pH 6 and requires acid to ensure the protonation of the primary amine. Its solubility is dependent on the deacetylation (and thereby the pKa value of the chitosan) and the pH. Chitosan with 40% of deacetylation is soluble up to pH 9, whereas chitosan with about 85% of deacetylation is soluble only up to pH 6.5 [10]. Additionally, it is slightly soluble in organic solvents, such as dimethyl sulfoxide (DMSO) and *p*-toluene sulfonic acid [14]. This poor solubility is a limitation for the processing of chitosan and its chemical modification. Therefore, various chemical modifications (i.e., quaternization, and grafting succinic acid or ethylene glycol) have been introduced to increase the water solubility of chitosan [8]. GC is a chitosan derivative conjugated with hydrophilic ethylene glycol branches, and it is water soluble at a neutral/acidic pH where the pendent ethylene glycol branches on polymer increase both aqueous solubility of the native chitosan and provide steric stabilization [15]. Additionally, the presence of reactive functional groups, including amine and hydroxyl groups in the GC backbone, offers flexibility for various chemical modifications [16]. Through numerous synthetic strategies, GC can be easily modified to afford a large number of derivatives for drug delivery. These GC derivatives form self-assembled nanostructures and can be used as drug delivery systems to carry therapeutic drugs and diagnostic agents [5]. In this review, we summarize the versatile chemical modification methods used to prepare various GC derivatives (e.g., various GC derivatives as drug carriers, GC-drug conjugates using a crosslinker, specific receptor-targeted GC, and stimuli-responsive GC) and describe the recent progress in GC-based drug delivery carriers for disease therapy.

## 2. Versatile Chemical Derivatizations of Glycol Chitosan

### 2.1. Preparation of GC

GC is synthesized by the incorporation of the hydrophilic glycol group, which introduced by reacting chitin with ethylene oxide followed by its deacetylation [17]. Knight et al. have performed ^1^H-NMR characterization of the purified GC purchased from Sigma-Aldrich to determine the degree of acetylation. The degree of acetylation was calculated to range from 10 to 13% in the purified GC samples. The remaining 87 to 90% would be the total contribution of the primary, secondary, and tertiary amine groups. Depending on the extent of deacetylation, GC contains 5 to 8% nitrogen, which is mostly in the form of primary amine groups [18]. The number average (Mn) and weight average (Mw) molecular weight of GC are 1.71 × 10^5^ and 1.95 × 10^5^, respectively. Depolymerization of GC with nitrous acid reduced the molecular weight of GC up to approximately 7 × 10^3^. This depolymerization can alter its chemical structure, as the secondary amine groups were converted to potentially carcinogenic *N*-nitrosamines [17]. However, no difference in cytotoxicity was observed with varying GC molecular weight, indicating the biocompatibility of GC is not lost via reduction in its molecular weight. More importantly, a water soluble GC has the free amine groups along the backbone, allowing for further modification or interaction with the host cells. These facts imply that GC is a suitable material for various pharmaceutical and biomedical applications. 

### 2.2. Design of GC Derivatives as Drug Carriers

Amphiphilic or hydrophobically-modified polymers have been extensively investigated in the field of nanobiotechnology and pharmaceuticals. In general, amphiphilic polymers can be readily prepared by conjugating hydrophilic polymers with hydrophobic molecules through various chemical reactions because GC has a large number of amine and hydroxyl groups on its backbone. Amphiphilic polymers self-assemble in aqueous solution because they are composed of an inner hydrophobic core and an outer hydrophilic shell. Additionally, the prepared amphiphilic GC derivatives can easily incorporate various types of drugs (e.g., hydrophobic anticancer drugs, peptides, or nucleic acids) within the nanoparticles via hydrophobic/electrostatic interactions or intermolecular crosslinking [19,20,21,22,23,24]. 

#### 2.2.1. Hydrophobically-Modified GC (HGC) Derivatives for Anticancer Drug Delivery

**Figure 1:**
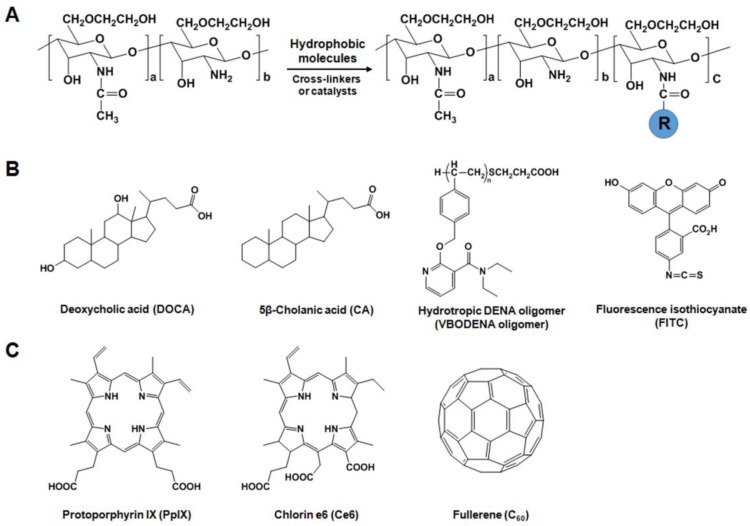
(**A**) General preparation methods of hydrophobically modified glycol chitosan (HGC) derivatives. HGC derivatives were chemically modified with various hydrophobic molecules in the presence of crosslinkers or catalysts. R means hydrophobic moieties. (**B**) Examples of hydrophobic molecules for preparation of HGC-based drug carriers. (**C**) Several photosensitizers such as PpIX, Ce6 or C_60_ can be coupled to GC for photodynamic therapy.

HGC derivatives are prepared by chemical conjugation of hydrophilic GC with hydrophobic molecules (Figure 1A). Introduction of hydrophobic molecules such as deoxycholic acid (DOCA), 5β-cholanic acid (CA), and hydrotropic DENA oligomers (VBODENA oligomers) into the GC polymer led to the formation of self-aggregated nanoparticles (Figure 1B). To synthesize HGC derivatives, the amine groups of GC (Mw = 2.5 × 10^5^, degree of deacetylation = 82.7%) were reacted with the carboxylic groups of DOCA, CA, or VBODENA oligomers using EDC (1-ethyl-3-(3-dimethylaminopropyl)carbodiimide) and *N*-hydroxysuccinimide (NHS) or HOBt (1-hydroxybenzotriazole) [25,26]. To prepare GC-CA conjugates, GC was dissolved in distilled water, followed by dilution with methanol. Then, GC was chemically reacted with CA by adding equal amounts of EDC and NHS. The resulting solution was dialyzed and freeze-dried to obtain GC-CA conjugates. The freshly synthesized GC-CA conjugates have 150 ± 4.5 moles of CA per one GC chain (GC-CA_150_). Due to their hydrophobic inner cores, GC-CA_150_ conjugates can easily imbibe various anticancer drugs such as paclitaxel (PTX) [21,26,27], cisplatin [28], camptothecin (CPT) [29], docetaxel (DTX) [30], and peptide [23]. Importantly, the use of GC-CA_150_ conjugates can solve the solubility problems of PTX [21] and DTX [30], as well as protect CPT from hydrolysis [29]. GC-VBODENA conjugates were synthesized by chemical reaction of GC (Mw = 2.5 × 10^5^, degree of deacetylation = 82.7%) with VBODENA oligomers (Mn = 3520). GC was dissolved in distilled water, and VBODENA ologimers dissolved in methanol was added under stirring. The chemical reaction was initiated by adding equal amounts of EDC and HOBt. The conjugation of hydrotropic VBODENA oligomers to GC can increase drug loading content (or efficiency) by almost two-fold compared to GC-CA_150_ nanoparticles because hydrotropic oligomers are water-soluble compounds that can enhance the water solubility of sparingly soluble drugs [26,27]. By further conjugating the fluorescent reactive dye Cy5.5-NHS (cyanine 5.5-NHS ester) to GC-CA_150_ nanoparticles and using bioimaging equipments, their blood circulation time, preferential accumulation in tumor tissues, and drug injection intervals could be determined [28,29,30]. Based on the information obtained regarding the pharmacokinetics, biodistribution, and injection frequency of the Cy5.5-labeled GC-CA_150_ (Cy5.5-GC-CA_150_) nanoparticles, they were found to exert potent therapeutic efficacies against several cancers including melanoma (B16F10) [23], squamous cell carcinoma (SCC7) [26,28], human lung carcinoma (A549) [29] and human breast adenocarcinoma (MDA-MB-231) [27,30]. Another hydrophobic molecule, fluorescence isothiocyanate (FITC) can be reacted with amine groups of GC in 0.5 M sodium carbonate buffer to yield GC-FITC nanoaggregates (234 nm in PBS), which allow analysis of their biodistribution in tumor-bearing mice [19,20]. Smaller molecular weight (Mw = 6.7 × 10^4^, degree of deacetylation = 88%) GG-FITC molecules (~20 nm) could successfully visualize the lipid rafts (<50 nm) in live cells [31]. 

#### 2.2.2. GC Derivatives for Nucleic Acid Delivery

To achieve efficient systemic delivery of nucleic acids, non-viral vehicles have been developed because they are nontoxic and less immunostimulatory. Recently, small interfering RNA (siRNA) has attracted attention as a potential therapeutic due to its highly sequence-specific gene silencing ability and generality of targets. Due to the degradation by nuclease, however, it is difficult to achieve systemic delivery of siRNA to exert its therapeutic effects. Positively-charged GC-based nanoparticles have been tested for the systemic delivery of nucleic acids such as siRNA. However, the interactions between GC-CA_150_ nanoparticles and siRNA are too weak to form condensed and stable nanoparticles. To enhance the positive charge of the nanoparticles, polyethyleneimine-CA (PEI-CA) conjugates were synthesized through the chemical reaction of PEI (branched, Mw = 2.5 × 10^4^) and CA in the presence of HSPyU (dipyrrolidino (*N*-succinimidyloxy carbenium hexafluorophosphate)) [32]. PEI-CA conjugates have 7 ± 1.5 moles of CA per one PEI chain (PEI-CA_7_, Mw = 2.8 × 10^4^). To obtain more stable and condensed nanoparticles, GC-CA_150_ (ζ = 10.8 mV) and PEI-CA_7_ were mixed together at a 1:1 weight ratio to yield GC-PEI nanoparticles (size = 350 nm, ζ = 23.8 mV). The complexes of siRNA (RFP) and GC-PEI at a 1:5 weight ratio formed more stable nanoaggregates (size = 250 nm, ζ = 9.95 mV) with nearly 100% loading efficiency due to the increased positive charge caused by mixing GC-CA_150_ and PEI-CA_7_. Additionally, by protecting siRNA (RFP) from nuclease degradation and effectively delivering siRNA (RFP) to the cytoplasm and tumor tissues, siRNA (RFP)/GC-PEI nanoparticles exerted a remarkable silencing effect on RFP expression in RFP-B16F10 tumor cells or tissues. To successfully deliver plasmid DNA (pDNA) into the nucleus of cells, Lee et al. synthesized GC-methyl acrylate-PEI (GMP) as a non-viral gene delivery vector by grafting PEI (Mw = 800) onto GC (Mw = 4.9 × 10^5^, degree of deacetylation = 91.6%) via amidation after Michael addition using methyl acrylate [33]. GMP can strongly interact with pDNA via electrostatic interaction to form GMP/pDNA complexes, which have positively-charged rod-like shapes (size = 400 nm, ζ = 30 mV). GMP had relatively low cytotoxicity and high transfection efficiency in human epithelial ovary carcinoma (HeLa), human embryonic kidney 293 (HEK293), and human hepatocellular liver carcinoma (HepG2) cells, in comparison to high molecular weight PEI (Mw = 2.5 × 10^4^). They demonstrated that GMP polymer could efficiently transfer pDNA (Enhanced Green Fluorescent protein-C2 (EGFP-C2)) into human adipose-derived mesenchymal stem cells (AD-MSCs), and showed GMP polymer did not disrupt the characterization of human AD-MSCs following cell penetration [34]. 

#### 2.2.3. GC Derivatives for Photodynamic Therapy (PDT)

For the application of GC derivatives in PDT, a hydrophobic photosensitizer (PS) can be loaded into nanoparticles or chemically-coupled to water soluble polymers (Figure 1C). The Kwon group developed protoporphyrin IX (PpIX)- or chlorin e6 (Ce6)-loaded GC-CA_150_ nanoparticles that are nano-structures with approximately 300 nm in diameter [35,36]. These PpIX- or Ce6-loaded GC-CA_150_ nanoparticles showed more effective PDT in vitro and in vivo than free PpIX or Ce6. However, the physical encapsulation of PpIX or Ce6 into nanoparticles showed burst release from nanoparticles due to their instability in the blood circulation [35,36]. This problem can be overcome by direct chemical conjugation of GC (Mw = 2.5 × 10^5^, degree of deacetylation = 82.7%) with Ce6 using EDC and NHS [36]. Compared to Ce6-loaded GC-CA_150_, no burst release of Ce6 from the GC-Ce6 nanoparticles (approximately 250–300 nm) was observed, leading to less phototoxicity in vitro. However, GC-Ce6 had a prolonged circulation time and accumulated more specifically in tumor tissues, resulting in better PDT effects than Ce6-loaded GC-CA_150_ nanoparticles. As another example of PS, fullerene (C_60_) has been used as a potentially photoactivatable agent for PDT in biological systems because it can generate reactive oxygen species (ROS) under visible light irradiation [37,38]. However, due to its inherent extreme hydrophobicity and tendency to aggregate in water and biological media, C_60_ itself is less promising as a photoactivatable drug in biomedical applications [37]. This shortcoming can be resolved by chemical conjugation between the free amine groups of GC (Mw = 5.0 × 10^5^) and the C=C double bonds of C_60_ in anhydrous benzene/DMSO containing triethylamine (TEA) [39]. Compared to unconjugated C_60_, GC-C_60_ conjugates (approximately 10–23 nm) increased the solubility and light-sensitivity of C_60_, leading to significant cell death of KB cells. 

The bioorthogonal chemical reporter system is a novel method to label and visualize biomolecules in vivo without genetic manipulation [40]. In this approach, metabolic labeling of the cell membranes with azide groups primes the target biomolecule. As a chemical reporter, the azide group is widely used due to its small size, metabolic stability, and lack of reactivity with natural biofunctionality [41]. Among various alkyne compounds, cyclooctynes react with azides without copper to achieve bioorthogonal labeling [42]. Therefore, copper-free click chemistry between azides and cyclootynes has been widely used in biological and biomedical fields to label proteins, nucleotides, or cells and to modify nanoparticle surfaces [43,44,45,46]. Recently, Lee et al. suggested a novel two-step PDT approach in vivo using both metabolic glycoengineering and copper-free click chemistry [47]. First, effective delivery and rapid uptake of the precursor Ac4ManNAz (tetraacetylated *N*-azidoacetyl-d-mannosamine)-loaded GC-CA_150_ nanoparticles into tumor cells or tumor-bearing mice generated site-specific azide groups on tumor cells or tissues irrespective of the type of tumor cell (human oropharyngeal carcinoma (KB), A549, human glioblastoma (U87MG), human breast adenocarcinoma (MCF-7), and human breast carcinoma (MDA-MB-468, and MDA-MB-436)). Second, to more specifically deliver photoactivatable agents to the azide groups generated on tumor cells or tissues, bicycle [6.1.0] nonyne *N*-hydroxysuccinimide ester II (BCN-NHS) was conjugated with GC (Mw = 2.5 × 10^5^, degree of deacetylation = 82.7%) in DMSO/distilled water for overnight to yield BCN-GC. Then, BCN-GC was reacted with Ce6 in the presence of EDC and NHS. About 39 molecules of Ce6 and 37 molecules of BCN were conjugated to one GC polymer to BCN-GC-Ce6 nanoparticles (300 nm). Through copper-free click chemistry, BCN-GC-Ce6 nanoparticles specifically bound to azide groups on tumor cells and accumulated more in tumor tissues after the generation of azide groups by pretreatment with Ac4ManNAz-loaded GC-CA_150_. Additionally, in vivo studies demonstrated that a two-step approach using Ac4ManNAz-loaded GC-CA_150_ and BCN-GC-Ce6 nanoparticles showed more effective tumor destruction after laser irradiation compared to free Ce6 or BCN-GC-Ce6 nanoparticles alone. 

### 2.3. Specific Receptor Targetable GC Derivatives

Most nano-sized drug carriers can penetrate tumor vasculatures and preferentially accumulate at tumor tissues through the leaky endothelium [48]. However, they often do not accumulate in tumor tissues with a poorly developed vasculature [49]. This problem can be overcome by chemical modification of the specific targeting moieties on the surface of the nanoparticles. Additionally, conjugation of specific targeting ligands to the nanoparticles can improve therapeutic and diagnostic capabilities of the drugs or imaging agents because the targeting ligands can specifically recognize and interact with target molecules expressed on the diseased tissues [50,51]. 

**Figure 2:**
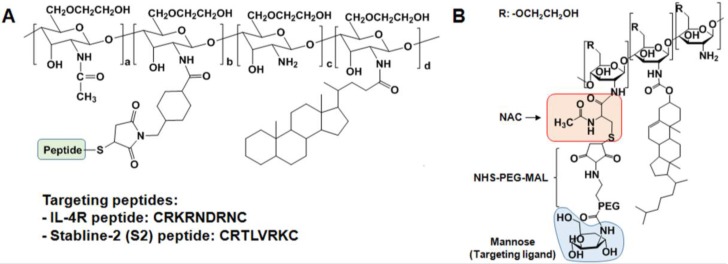
Specific receptor targetable GC derivatives. (**A**) Specific receptor targetable peptides such as IL-4R- and S2-peptide can be coupled to GC-CA_150_ nanoparticles using SMCC as a crosslinker. (**B**) Chemical structure of macrophage mannose receptor-targeting nanoparticles (MMR).

As described above, the binding abilities of the nanoparticles to specific receptors can be improved by tagging the surface of nanoparticles with peptides that target a specific receptor. These specific receptor binding peptides can be discovered and selected using the phage display technique, which is a very useful method to select peptides with specific binding affinities from a large number of variants and a promising approach to develop novel targeted drug delivery systems and molecular imaging agents to evaluate interactions between proteins and ligands in vitro and in vivo [52,53,54]. Through the phage display method, the Kim group discovered atherosclerotic plaque-targeted peptides such as CRKRLDRNC (termed the interleukin 4-receptor (IL-4R) peptide) and CRTLTVRKC (termed the stabline-2 (S2) peptide). The IL-4R and S2 peptides can selectively bind to IL-4R and S2, respectively, which are expressed on endothelial cells, macrophages, and smooth muscle cells in atherosclerotic plaques (Figure 2A) [53,55]. GC-CA_150_ conjugates were reacted with SMCC (4-(*N*-maleimidomethyl)cyclohexane carboxylic acid *N*-hydroxysuccinimide ester). Then, the thiol group on the peptides was coupled to the maleimide group of the SMCC-conjugated GC-CA_150_ to prepare the peptide-tagged GC-CA_150_ nanoparticles. In vivo studies demonstrated that IL-4R- or S2-peptide-tagged GC-CA_150_ nanoparticles have high binding affinity to atherosclerotic lesions. These studies also found that IL-4R peptide-tagged nanoparticles containing PTX selectively targeted IL-4R overexpressing human lung squamous carcinoma (H226) cells and tissues, resulting in significant cell death and prevention of H226 tumor growth [54]. 

Macrophages are pivotal contributors to plaque destabilization through the release of various inflammatory precursors (e.g., proteases, ROS, and immune mediators) [56,57]. Therefore, they have emerged as key target biomarkers for high-risk coronary atheromata [58,59,60]. Recently, Kim et al. developed a macrophage mannose receptor (MMR)-targeted near-infrared fluorescence (NIRF) nanoprobe to specifically target macrophages in high-risk plaques (Figure 2B) [61]. The MMR-targeting nanoprobe was prepared as follows: GC (Mw = 2.5 × 10^5^, degree of deacetylation = 82.7%) was reacted with NAC (*N*-acetylcysteine) in MES (4-morpholineethanesulfonic acid sodium salt) buffer (pH 5.6) containing EDC and NHS to yield thiolated GC (tGC). Then, to endow targetability to mannose receptors, the thiol group of tGC was reacted with MAN-PEG-MAL (mannose-polyethyleneglycol-maleimide) in PBS buffer (pH 6.9). The MAN-PEG-GC derivative was further reacted with cholesteryl chloroformate (Chol) and Cy5.5-NHS ester in DMSO:DMF cosolvent to obtain Cy5.5-labeled MAN-PEG-GC-Chol (termed the MMR nanoprobe). This MMR nanoprobe showed high affinity to mannose receptors and allowed the direct visualization of plaque macrophages in carotid plaques. Furthermore, the MMR nanoprobe facilitated in vivo intravascular imaging of plaque inflammation in coronary-sized vessels of atheromatous rabbits using an optical coherence tomography (OCT)-NIRF catheter-based imaging system. This MMR nanoprobe has a hydrophobic inner core since it contains hydrophobic cholesterol moieties. Thus, this amphiphilic structure of the MMR nanoprobe will be useful to deliver antiplaque drugs. 

### 2.4. Endogenous Stimuli-Responsive GC Derivatives

Nano-sized drug delivery systems are highly effective in enhancing the therapeutic efficacies of drugs while reducing their side-effects [1]. Although various nano-sized drug delivery systems have shown promising results in experimental and preclinical animal models, the translation of nano-sized drug delivery systems into the clinic is still questionable due to inferior pharmacokinetics, premature drug release into the blood circulation, unwanted accumulation in normal tissues, poor tumor penetration capacity, and uncontrollable drug release at target sites [62,63]. To resolve these drawbacks, nano-sized drug delivery systems have been prepared using stimuli-responsive materials, which can be sensitive to a variety of endogenous stimuli, such as acidity, redox potential (glutathione (GSH: γ-glutamyl-cysteinyl-glycine tripeptide)), enzymes, ROS, and hypoxia [62,63]. These endogenous stimuli can trigger the release of drugs from delivery carriers at specific sites of diseased cells or tissues. Therefore, stimuli-responsive drug carriers can enhance therapeutic effects, as well as reduce side effects of drugs. 

#### 2.4.1. pH-Sensitive GC Derivatives

pH variations have been examined for the ability to trigger the release of drugs when subtle environmental changes occur in cancer or inflammation. An acidic extracellular or intracellular pH has been considered as an appropriate endogenous trigger for the controlled release of drugs in tumor tissues and/or within endosomes and lysosomes [63]. Compared to pH values in the blood and normal tissues (pH 7.4), extracellular pH values in tumors range from 6.0 to 7.2 [64], and the intracellular pH is decreased to 5.0–6.0 in endosomes and 4.0–5.0 in lysosomes [65,66]. 

**Figure 3:**
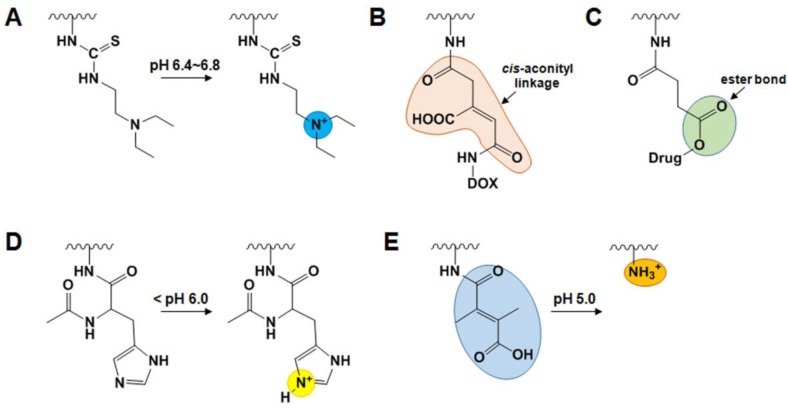
pH sensitive GC derivatives. (**A**) Self-assembled GC-DEAP in neutral pH drastically underwent conformational changes at acidic extracellular pH via the dissociation after protonation of DEAP moiety in GC. (**B**,**C**) Polymer-drug conjugates which were linked through *cis*-aconityl linkage or ester linkage were cleaved at acidic intracellular pH environment, leading to release of drugs into the cytosol. (**D**) Under endosomal pH (below pH 6.0), the imidazole group of NAcHis in NAcHis-GC derivatives were protonated to destabilize the self-assembled nanostructures of NAcHis-GC. (**E**) DMA (2,3-dimethylmaleic anhydride) moiety conjugated to GC was stable at neutral pH. Once it is exposed to acidic endosomal pH 5.0, the amides between GC and DMA were hydrolyzed to regenerate the positively charged amine group.

In response to variations in the acidic extracellular pH, nano-sized drug carriers with ionizable groups undergo conformational and/or solubility changes, leading to a fast release of drugs from the nanoparticles to the tumor tissues. The Lee group developed a smart pH-sensitive GC nanogel and photoactivatable nanoagents for extracellular pH targeted drug delivery in tumors [67,68]. This smart pH-sensitive system was prepared through the chemical reaction between the isothiocyanate group of the pH sensitive moiety DEAP (3-diethylaminopropyl isothiocyanate) and the primary amine groups of GC (Mw = 2.5 × 10^5^, degree of deacetylation = 82.7%) in DMSO containing TEA and pyridine [67]. GC-DEAP conjugates formed self-assembled nanogels with mean diameters of 102 nm at neutral pH due to the hydrophobic nature of DEAP. However, once GC-DEAP nanogels become protonated, ionized GC-DEAP undergoes a drastic conformational change from a nanogel to a soluble polymer at the extracellular of pH 6.8 (Figure 3A), leading to fast release of the encapsulated doxorubicin (DOX). To develop smart pH-sensitive photoactivatable nanoagents, they further reacted the amine groups of GC-DEAP with both Ce6 and PEG-COOH in the presence of DCC (*N*,*N*′-dicyclohexylcarbodiimide) and NHS [58]. Their self-assembled nanostructures (approximately 150 nm in diameter) were switched into soluble molecules upon exposure to the extracellular acidic pH (pH 6.8 or 6.4). Additionally, laser irradiation of the soluble molecules induced remarkable cell death in vitro and effectively destroyed HeLa tumor masses in vivo by generating singlet oxygen.

For intracellular drug delivery, pH-sensitive drug carriers have been developed by designing polymer/carrier-drug conjugates with pH-sensitive moieties. Acid-sensitive moieties are stable at neutral pH, but can be cleaved at acidic intracellular pH, thus enabling the release of drugs by degrading the polymer/carrier-drug linkage or by disrupting the nanocarriers via modification of the charge of the polymer [62,63]. For example, DOX-conjugated GC (DOX-GC) with a *cis*-acotinyl spacer was synthesized through two successive reaction steps (Figure 3B) [19]. The amine group of DOX was coupled to the carboxyl group of *cis*-aconitic anhydride in dioxane containing. Then, *N*-*cis*-aconityl DOX was further conjugated to the amine groups of GC (Mw = 2.5 × 10^5^, degree of deacetylation = 82.7%) using EDC and NHS. DOX-GC conjugates with DOX content in the range of 2–5 wt% self-assembled in aqueous solution. Additionally, due to the hydrophobic nature of DOX, additional DOX could be physically entrapped in the nanoparticles. The release of DOX from the nanoparticles was accelerated at pH 4 because the *cis*-aconityl spacer is readily cleavable at low pH. Ginsenoside compound K (CK)-conjugated GC (CK-GC) was also synthesized via a two-step chemical reaction of GC (Mw = 4.3 × 10^5^, degree of deacetylation = 75.2%) and CK using succinic anhydride [69]. Carboxylated CK (CK-COOH) was coupled through an ester bond by reacting CK with succinic anhydride in pyridine/dichloromethane. Then, chemical conjugation of CK-COOH and GC was done in distilled water/methanol containing EDC and NHS. Stable and self-assembled CK-GC nanoparticles at pH 7.4 became highly unstable at the acidic pH 5.0 due to the acid cleavage of ester bonds in the succinate linker (Figure 3C). Fast release of CK from CK-GC nanoparticles at acidic pH exhibited high cytotoxicity in HT29 (human colon adenocarcinoma) and HepG2 cells. For efficient intracytoplasmic delivery of anticancer drugs to eradicate cancer cells, *N*-acetyl histidine-conjugated GC (NAcHis-GC) has also been developed [70,71]. To prepare NAcHis-GC, the different amounts of NAcHis (with an imidazole group, pKa value of 6.5, and pH-responsive fusogen) was activated using EDC and NHS, and the activated NAcHis was further reacted with the amine groups of GC (Mw = 2.5 × 10^5^, degree of deacetylation = 82.7%). The self-assembled nanostructures of NAcHis-GC (150–250 nm in size) at neutral pH were destabilized when the imidazole group of NAcHis became protonated under endosomal environments (Figure 3D), leading to release of the encapsulated drugs (such as PTX and DOX) into the cytosol. Recently, the Lee group developed an endosomal pH-activated GC-DMA-C_60_ derivative for PDT [72]. GC-DMA-C_60_ derivatives were synthesized as follows: GC (Mw = 5.0 × 10^5^) were reacted with DMA (2,3-dimethylmaleic anhydride) in DMSO containing TEA and pyridine. Next, the free hydroxyl groups of GC-DMA were coupled to the π-π carbon bonds of C_60_ in toluene/DMSO containing lithium hydroxide (LiOH). The GC-DMA-C_60_ formed self-assembled multi-nanogel aggregates (283 nm in size) at neutral pH due to electrostatic interactions between the carboxyl groups of pendant DMA and the residual primary amine groups of GC. Multi-nanogel aggregates of GC-DMA-C_60_ showed no noticeable singlet oxygen generation upon light irradiation at 670 nm. However, in an acidic environment, the amide bond between GC and DMA hydrolyzed to regenerate the positively-charged amine groups at endosomal pH 5.0 (Figure 3E), followed by the multi-nanogel aggregates converting to single nanogel status (approximately 46 nm in size) due to electrostatic repulsions. This conversion from multi-nanogels to single nanogel induced phototoxicity, leading to significant cancer cell death. 

#### 2.4.2. GSH-Sensitive GC Derivatives

As an endogenous reducing agent, GSH is the most abundant thiol in mammalian cells and plays an important role in major biological functions [73]. In general, GSH is present at extremely low concentrations of 2–20 μM in the blood or extracellular compartments, whereas its concentration is as high as 0.5–10 mM in the intracellular milieu and in tumor tissues compared with healthy tissues [74,75]. Disulfide bonds are stable in the blood and the extracellular compartment at low levels of GSH, but are rapidly cleaved by GSH in a highly reducing intracellular environment [62]. This effect of increasing the GSH concentration can be used to attain redox sensitivity and achieve cytosolic drug release by reductive degradation of drug carriers [76]. 

**Figure 4:**
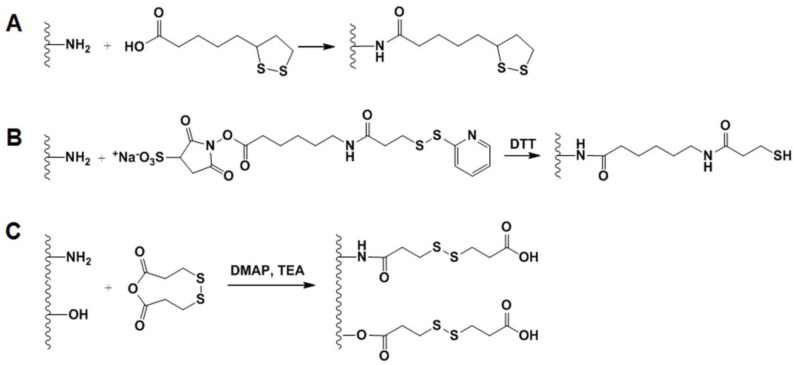
(**A**) Synthetic schemes for preparation of GC-lipoic acid (GC-LA). Due to hydrophobic property of LA, GC-LA can encapsulate hydrophobic drug and further core-crosslinking using DTT. (**B**) Thiolated GC (tGC) derivative was prepared by conjugating GC with Sulfo-LC-SPDP, followed by treating DTT. The tGC was further cross-linked to various poly-siRNA with dithiol end groups. (**C**) To synthesize bioreducible PEI-GC as a gene delivery carrier, DTDPA was conjugated to GC through amide or ester bonds in the presence of DMAP and TEA.

Recently, a bioreducible disulfide linked pheophorbide A-GC (PheoA-ss-GC) conjugate was synthesized as a potential PS carrier for PDT [77]. First, the carboxyl group of PheoA was pre-activated in DMSO containing EDC and NHS, and cysteamine was then reacted with pre-activated PheoA to yield PheoA-cysteamine. The synthesized PheoA-cysteamine dissolved in DMSO was reduced using DTT (dithiothreitol) under nitrogen gas to obtain thiolated PheoA. Second, GC (Mw = 2.5 × 10^5^, degree of deacetylation = 82.7%) was reacted with SPDP (*N*-succinimidyl 3-(2-pyridyldithio) propionate) in DMSO to obtain GC-ss-Py. Finally, PheoA-ss-GC was prepared by reacting the thiolated PheoA with GC-ss-Py in DMSO under a nitrogen atmosphere. PheoA-ss-GC self-assembled to form spherical nanoparticles about 200 nm in size. Significant photoactivity of PheoA-ss-GC nanoparticles was observed under an intracellular reductive environment due to cleavage of the disulfide bonds. Furthermore, in vivo studies showed that PheoA-ss-GC nanoparticles selectively accumulated at the tumor sites and prolonged circulation, resulting in significant inhibition of tumor growth. More recently, Zhou et al. developed GSH-responsive core-crosslinked nanocarriers for intracellular DOX delivery into A549 cancer cells as follows [78]: GC (Mw = 4.3 × 10^5^, degree of deacetylation = 75.2%) was chemically conjugated with lipoic acid (LA) in the presence of EDC (Figure 4A). Then, DOX was encapsulated into GC-LA nanoparticles via hydrophobic interactions between DOX and LA. Finally, core-crosslinked DOX-loaded GC-LA (DOX/GC-LA/cc) nanoparticles were obtained by further stirring of DOX-encapsulated GC-LA nanoparticles in borate buffer (pH 8.4) containing DTT for 24 h under nitrogen gas at 37 °C. DOX/GC-LA/cc nanoparticles released only approximately 25.3% of the total DOX over 96 h in PBS (pH 7.4) without GSH, whereas they released up to almost 100% of the DOX over 96 h in PBS containing 20 mM GSH (the intracellular GSH level in cancer cells). This significant increase in drug release might be due to the cleavage of polydisulfide crosslinking, which strengthens the nanoparticle structures and allows the crosslinked carriers to better reserve hydrophobic drugs within the core of drug carriers. 

Systemic delivery of siRNA is limited in clinical applications because of its susceptibility to degradation by nuclease. To resolve this issue, the Kim group developed an alternative siRNA, polymerized siRNA (poly-siRNA), to enhance siRNA stability [79]. To synthesize poly-siRNA, dithiol-modified siRNA (RFP or VEGF siRNA) bearing thiol groups at the 5′-ends of both sense and anti-sense strands were crosslinked using *N*,*N*,*N*′,*N*′-tetramethyl-azodicarboxamide. These poly-siRNAs (RFP or VEGF) were degraded by serum nuclease within 12 h, whereas mono-siRNAs were degraded within 1 h, suggesting that poly-siRNA is much more stable than mono-siRNA [80]. Despite the higher stability of poly-siRNA than mono-siRNA, the degradation of poly-siRNA is still problematic due to enzymatic degradation in the blood stream. To overcome this problem, tGC polymer was synthesized by reacting GC (Mw = 2.5 × 10^5^, degree of deacetylation = 82.7%) with Sulfo-LC-SPDP (sulfosuccinimidyl-6-[3′-(2-pyridyldithio)-propionamido] hexanoate) in PBS buffer (pH 7.4) and reducing with DTT (Figure 4B) [80]. Through charge-charge interaction and chemical self-crosslinking between tGC and poly-siRNA, the tGC polymer formed stable nanoparticles with poly-siRNA (RFP or VEGF), and the stability in serum was increased to 24 h. After systemic administration, poly-siRNA (RFP or VEGF)-tGC nanoparticles showed effective RFP gene silencing or significant inhibition of neovascularization in vivo, leading to effective tumor suppression of RFP-SCC7 tumors compared to poly-siRNA (RFP or VEGF) and poly-siRNA (RFP or VEGF/PEI polyplexes). Additionally, this tGC polymer could efficiently deliver single-gene or dual-gene targeted poly-siRNAs, such as poly-siRNA (TNF-α) or dual-poly-siRNA (VEGF/BCl-2), and showed effective therapeutic results in rheumatoid arthritis or human prostate cancer (PC3) tumors [81,82]. The combination treatment of poly-siRNA (P-glycoprotein) and DOX exerted synergistic anti-tumor effects on DOX-resistant MCF-7 tumors by overcoming multi-drug resistance (MDR) [83]. Additionally, systematic co-delivery of poly-siRNA (VEGF)-tGC and bevacizumab showed improved therapeutic efficacy in human squamous carcinoma (A431) tumors [84]. More recently, they newly developed another type of polymeric siRNA RAPSI (referred to ‘rolling circle transcriptin (RCT) and annealing-generated polymeric siRNA’) nanoflower using a unique extended form of RCT method (firstly, amplifying antisense strands of siRNA via RCT process and, secondly, annealing its chimeric RNA-DNA sense strands), as an RNAi therapeutics platform technology [85]. The synthesized RAPSI product with a RNA multi-layered nanoflower structure (diameter: approximately 1 μm). The RAPSI nanoflower could be successfully condensed with tGC to form RAPSI/tGC nanoparticles (approximately 240 nm) because sulfhydryl groups in tGC promote particle condensation and stabilization. The formed RAPSI/tGC nanoparticles were accumulated specifically in PC3 tumor tissues, and they released biological active form of siRNA monomers in the cytoplasmic region, resulting in the sequence-specific gene silencing and remarkable tumor growth inhibition with VEGF-targeted siRNA. 

The Hourigan group developed bioreducible PEI-functionalized GC (GC-ss-PEI) derivatives to facilitate intracellular gene release along with excellent redox-responsive characteristics [86]. GC (Mw = 2.5 × 10^5^) was reacting with 3,3-dithiodipropionic anhydride (DTDPA) in the presence of dimethylaminopyridine (DMAP) by dropping and further adding TEA to obtain GC-ss-COOH (Figure 4C). Then, GC-ss-COOH was preactivated with EDC and NHS, and the mixture was reacted with low molecular weight PEI (Mw = 2.5 × 10^3^) to achieve GC-ss-PEI. The GC-ss-PEI expressed good DNA binding efficiency via electrostatic interactions to form nanoparticles (size = 50–90 nm), and redox-responsive characteristics. Moreover, the transfection efficiency of GC-ss-PEI was higher than for high molecular weight PEI (HMW PEI, Mw = 2.5 × 10^4^) against HEK293 cells, while reducing cytotoxicity compared to HMW PEI.

### 2.5. External Stimuli-Sensitive GC Derivatives

Sustained drug release can also be achieved by externally-applied stimuli, including temperature changes, magnetic fields, ultrasound, light, and electric fields [62]. Although a number of external stimuli-responsive materials have been developed, there are few examples of external stimuli-responsive GC derivatives. As one example, the Yan group reported the development of dual pH-/UV light-responsive crosslinked polymeric micelles (CPM) [87]. The dual pH-/UV light-responsive CPM was synthesized by chemically coupling the amine group of GC (degree of polymerization = 2,800, degree of deacetylation = 82.7%) and the carboxyl group of NBS (*O*-nitrobenzyl succinate) in the presence of EDC and NHS and by further stabilizing through crosslinking GC-NBSC_19.3_ with glutaraldehyde (GA) to form GC-NBSC_19.3_ CPMs [87]. Similar to other amphiphilic polymers, GC-NBSC_19.3_ CPMs are able to self-assembly into nano-sized particles (approximately 37.8 nm) at pH 7.4 because of their amphiphilic structure comprised of hydrophobic NBS moieties and inter-crosslinking of GC with GA. When the pH was adjusted to 5.0 and kept for 5 h, the size of GC-NBSC_19.3_ CPMs increased to 71.2 nm, attributing to the cleavage of crosslinked imine bonds in the shells. When GC-NBSC_19.3_ CPMs is under pH 5.0 for 5 h and UV light irradiation (365 nm, 150 W) for 10 min, the particle sizes decreased to about 14.6 nm and no regular spherical nanostructures were observed, implying that light-sensitive NBS side groups were cleaved from the GC backbone under irradiation, causing disaggregation of the nanostructures. Furthermore, disaggregation of the nanostructures by UV light triggered the release of CPT from the nanoparticles, leading to better cytotoxicity against MCF-7 cancer cells compared to the non-irradiated nanoparticles. 

## 3. Conclusions

In this review, we described the versatile chemical modification methods that can be used to develop GC-based drug carriers for disease therapy. GC and its derivatives have attracted attention and have been extensively investigated for use in a wide range of biomedical applications because of their biocompatibility, biodegradability, and low toxicity. Additionally, due to the large number of reactive free amine and hydroxyl groups on the GC backbone, GC can be easily modified using various synthetic methods to afford a wide range of GC derivatives. GC derivatives can be synthesized via versatile chemical reactions of GC with various hydrophobic moieties (e.g., DOCA, CA, hydrotropic oligomers, and photosensitizers) and form self-assembled nanoparticles in aqueous media. Due to their amphiphilic properties and positive charge, they can readily encapsulate various kinds of drugs including anticancer drugs, peptides, nucleic acids, or photosensitizers. Compared to small drugs or soluble polymers, GC-based nano-sized drug carriers can more specifically deliver many types of drugs to diseased sites, leading to better therapeutic effects. Additionally, to improve the specific recognition and binding to diseased tissues, GC-based drug carriers can be further modified with specific targeting ligands, such as peptides or small molecular ligands. Moreover, many researchers have attempted to solve several of the drawbacks, including inferior pharmacokinetics, premature drug release in the blood stream, unwanted accumulation in normal tissues, and uncontrollable drug release at target sites by chemical modifications of GC with pH sensitive moieties, acid-labile bonds, disulfide bonds, or light-sensitive moieties. These endogenous or external stimuli-responsive GC derivatives can effectively deliver various drugs (e.g., anticancer drugs, siRNA, or poly-siRNA) by controlling drug release at specific diseased tissues rather than normal tissues. As described in this review, versatile chemical modifications of GC are able to produce various GC-based drug carriers or imaging agents with multi-functionality for the treatment of cancers, rheumatoid arthritis, or atherosclerosis. Except for the application of GC derivatives as drug carriers, recently, novel GC derivatives have still been developed for other biomedical applications. For example, an *N*-hexanoyl GC derivative as a thermo-reversible hydrogel can be useful as a convenient method for the development of in vitro 3D cell culture systems when used to coat the surfaces of cell culture dishes [88]. GC-EDTA (ethylenediaminetetraacetic acid) derivative was developed as an efficient metalloenzyme inhibitor to protect peptides and proteins from enzymatic degradation [89]. Conclusively, the development of novel GC derivatives using various synthetic approaches will be very helpful to design and optimize advanced and innovative drug carriers for personalized medicine.
